# Enhanced anaerobically digested swine wastewater treatment by the composite of polyaluminum chloride (PAC) and *Bacillus megatherium* G106 derived EPS

**DOI:** 10.1038/s41598-017-09044-0

**Published:** 2017-08-17

**Authors:** Junyuan Guo, Yang Huang, Cheng Chen, Yu Xiao, Jing Chen, Biyu Jian

**Affiliations:** College of Resources and Environment, Chengdu University of Information Technology, Chengdu, Sichuan 610225 China

## Abstract

A strain was isolated from biological sludge to produce EPS by using anaerobically digested swine wastewater (ADSW). Potential of the EPS in ADSW treatment were discussed. Results showed that the optimal fermentation medium for EPS production was determined as 4 g K_2_HPO_4_, 2 g KH_2_PO_4_, and 2 g sucrose dissolved in 1 L ADSW. After fermentation for 60 h, 2.98 g EPS with main backbone of polysaccharides can be extracted from 1 L of fermentation broth. The EPS showed good performances in ADSW treatment, after conditioned by this EPS, removal efficiencies of COD, ammonia, and TP reached 70.2%, 76.5% and 82.8%, respectively, which were higher than that obtained when chemicals were selected as conditioning agents. Removal efficiencies were further improved when the EPS and polyaluminum chloride (PAC) were used simultaneously, and finally reached 91.6%, 90.8%, and 92.5%, respectively, under the optimized conditioning process by the composite of EPS of 16 mg/L, PAC of 12 g/L, pH of 7.5, and agitation speed of 200 r/min.

## Introduction

Extensive swine breeding has already been highlighted as an effective way to increase meat production efficiency in China^1^, and thus, a large quantity of swine wastewater was generated^2^, which would adversely affect agricultural development and environmental quality as well as people’s lives if disposed without sufficient treatment^3^. Anaerobic digestion of livestock wastewater that converted organic matter into biogas allowed the production of renewable energy^4^, which has been vigorously promoted by China government. However, there still remained large amount of organic matters and ammonia after anaerobic process, which cannot be directly discharged without further treatment^5^. Ecological processes (e.g. artificial wetland) and advanced biochemical processes (e.g. SHARON-ANAMMOX) have been considered as the potential approaches to treat anaerobically digested swine wastewater (ADSW)^6^. However, practices proved that the above two methods were not efficient in the treatment of ADSW due to its poor biodegradability and low COD/NH_4_^+^-N ratio^7^.

Flocculation has been regarded as an easy and effective method in pretreatment of ADSW to improve the performance of ecological processes in China. Despite the economical and efficient benefits, the widely used chemical flocculants always posed serious health and environmental concerns^8, 9^. It is a fact that the composite flocculants can reduce the risk brought by chemical flocculants, since their doses can be decreased to the least, and several researches have tested and verified this fact. For example, Yang *et al*. optimized the flocculation conditions for kaolin suspension using the composite flocculant of MBFGA1 and PAC by response surface methodology^10^, Guo *et al*. enhanced dewatering of sludge with the composite of a bioflocculant and P(AM-DMC)^11^. In these two researches, the flocculating activity of kaolin suspension and the sludge dewatering ability were improved by using the composite, while the PAC and P(AM-DMC) doses were decreased, compared with their use alone. In this case, it is feasibility and meaningful to enhance the treatment of ADSW by the compound uses of chemicals and EPS.

In recent years, many researches have focused on the treatment of dying wastewater^12^, potato starch wastewater^13^, and heavy metal wastewater by extracellular polymeric substances (EPS)^14^. However, there were only one or two publications associating EPS and ADSW. EPS, produced by microorganisms, has attracted more and more attention due to its biodegradability, environment-friendly, and negligible secondary pollution^15, 16^. Currently, high costs associated with relatively expensive substrates (such as beef extract, peptone, and so on) have gradually become impediments for production and application of EPS^17^. Thus, attempts have been made to seek low-cost substrates (as cheap culture/fermentation medium), such as wastes, which rich in organic matter, nitrogen, and phosphorus, to reduce the production cost^18^. As known, there was large amount of organic matters and ammonia in ADSW, which could be a source for microorganisms’ growth and metabolism, and the metabolites could be a source to extract EPS. Therefore, microorganisms that can effectively utilize the substrates in ADSW to produce EPS are of academic and practical interests.

In this study, a microorganism was enriched from activated sludge by using LB (Luria Broth) medium. EPS extracted by this microorganism was applied to treat ADSW. Experiments first determined the EPS production process, active ingredient and characterizations. Subsequently, ADSW treated by various conditioning agents, including the EPS, FeCl_3_, Al_2_(SO_4_)_3_, and PAC, was compared. Furthermore, feasibility of the composite of the EPS and PAC in ADSW treatment was investigated by Response surface methodology (RSM). RSM, a statistical technique for building multivariable equation and evaluating their optimal values, can be effectively used to investigate the interactions of chemicals and EPS in the treatment of ADSW.

## Results

### Identification of the EPS-producing strain

An EPS-producing strain with a flocculating activity of 90.6%, named G106, was enriched from activated sludge by using LB medium with addition of 1000 mg/L of ammonia. Colony of this strain was circular, opalescent, and its surface was smooth on agar media. It is gram-positive, and some of physiological and biochemical characteristics were as follows: V-P test was negative, glycolysis and nitrate reduction test were all positive. In addition, this strain can grow well by using starch, gelatin, and citrate. 16 S rDNA gene sequence of this strain was registered in GenBank. Results showed that its 16 S rDNA sequence was highly similar with that of *Bacillus megaterium*, and the similarity reached 99.6%. According to 16 S rDNA gene sequence and physiological and biochemical characteristic, the strain G106 could be identified as *Bacillus megaterium*.

### EPS production and strain growth

Referred to the author’s previous publication^13^ about the optimization of fermentation process of *Paenibacillus polymyxa* in potato starch wastewater medium, this study investigated the effects of extra phosphate salts, nitrogen source, carbon source, and initial pH value of the ADSW medium on EPS production in turn, and the results were summarized in Figures. S1–S4. Figure. S1 depicted the EPS yield and its flocculating activity obtained from the ADSW medium (at its raw pH value of 6.7) with addition of different amounts of phosphate salts. Compared with the EPS yield of 0.65 g/L (0.65 g of purified EPS was harvested from 1 L fermentation broth) and flocculating activity of 63.2% from the phosphate-free medium after fermentation for 60 h, there was a significant improvement when the extra phosphate salt (K_2_HPO_4_:KH_2_PO_4_ = 2:1, *w*/*w*) were added into the ADSW medium, especially when the extra phosphate salt was adjusted to 6 g/L, both EPS yield and its flocculating activity reached their maximum of 1.62 g/L and 81.7%, respectively. In view of the fact that the phosphate itself almost has no flocculating activity, the above information illustrated that phosphate could promote EPS accumulation and activities during microbial metabolism^13^. From Figures. S2 and S3, all the selected carbon sources addition (including glucose and sucrose at their addition doses of 2 g/L, and methanol and 95% ethanol at their addition doses of 2 mL/L) can further promote the accumulation and activities of the EPS, especially when 2 g/L of sucrose was used as the optimal carbon source, EPS yield and its flocculating activity reached 2.98 g/L and 92.5% respectively, while extra inorganic and organic nitrogen sources addition (like (NH_4_)_2_SO_4_, NH_4_Cl, urea, and yeast extract at their doses of 2 g/L) almost have no promotion. A very important conclusion can be drawn from Figures. S1–S3 that the high-levels of ammonia (828 mg/L) in ADSW was enough for microorganism to produce EPS, while the carbon and phosphate source were not enough. The role of carbon addition during the bioflocculant production was similar with conclusions reported by Giri *et al*.^19^, in which glucose was the most preferred carbon source for *Bacillus subtilis*. From Figure. S4, it’s clearly showed that when adjusted initial pH of ADSW medium in the range of 5.5–8.5, the strain can effectively produce EPS, especially when the initial pH value was 7.5, EPS yield and its flocculating activity was appeared as 3.14 g/L and 95.8%, respectively. It is noteworthy that the pH range of 5.5–8.5 just covered the raw pH value of the swine wastewater (6.7), so that pH adjustment was not needed in this experiment from a practical standpoint. Similar researches reported that *Paenibacillus polymyxa* performed well under natural pH condition^14^, while a weak acidic was favored for *Streptomyces* ^20^.

The above findings reflected that the optimal fermentation medium for EPS production was determined as 4 g K_2_HPO_4_, 2 g KH_2_PO_4_, and 2 g sucrose dissolved in 1 L ADSW with initial pH value of 7.5, under this condition, the highest EPS yield and its flocculating activity were appeared as 3.14 g/L and 95.8%, respectively. From a practical standpoint, when the above ADSW medium’s pH value was maintained at its raw value of 6.7, EPS yield and its flocculating activity reached 2.98 g/L and 92.5%, respectively, which were decreased only 0.5% and 0.06%, respectively, indicated that ADSW can be used as an effective sources to produce EPS.

As seen from the strain’s growth curve in Figure. S5 over a fermentation time of 96 h, there was a rapid increasing in cell numbers of *Bacillus megaterium* G106 to 18.6 × 10^7^/mL at early stationary phase (60 h) which were maintained at a relatively stable level in the whole stationary phase (60–72 h). The EPS showed good correspondence to cell growth, with a highest value of 3.01 g/L achieved at 60 h, indicated that the EPS was formed during lag growth phase. The same phenomenon was observed in the cultivation of *Ochrobactrum ciceri* W2^18^. After the strain entered into its death phase at 72 h, cell number began to decline, and consequently the EPS yield decreased as well until to 1.26 g/L, which may be due to cell autolysis and the decreasing in enzymatic activity^14^. Additionally, the highest EPS value of 3.01 g was higher than that reported by Aljuboori *et al*.^17^ and Guo *et al*.^14^. For the medium’s pH value in Fig. S5, it was maintained at a constant level during 0–12 h, which might due to the buffering effects of phosphate salts, and then was decreased significantly from 6.7 to 4.6 within 60 h as the EPS yield increased, this may be ascribed to the producing and accumulating of organic acids during fermentation process, which were the main components of the EPS^21^. After entered into death phase, pH value was increased from 5.9 to 7.5, due to cell-lysis and the release of intracellular substances^14^.

### Characteristics of the EPS

Composition analysis revealed that the proportions of total sugar and protein contents were 92.8% and 6.1% (w/w), respectively. This data was similar with that reported by Wang *et al*.^18^. Further chemical analysis revealed that the total sugar mainly including 52.3% of neutral sugar, 23.5% of uronic acid, 22.6% of amino sugar, and so on, in addition, non-sugar components were almost negligible. The analyzed biopolymers from *Bacillus licheniformis* ^22^ was agreed with our observation, while the non-sugar components provided flexibility and stability for biopolymer MBF-UFH^23^. Furthermore, gel permeation chromatography indicated that the approximate molecular weight of the purified EPS was 4.21 × 10^5^ Da, a relatively high molecular weight, compared to the biopolymers produced by *Aspergillus flavus* ^17^ and *Agrobacterium* sp.^24^.

With regard to pH stability, the EPS was dissolved in a suitable volume of deionized water to achieve an initial flocculating activity of over 90% toward to 4 g/L kaolin clay suspension and divided into 9 aliquots, whose pH value was then adjusted to 3.5, 4.5, 5.5, 6.5, 7.5, 8.5, 9.5, 10.5, 11.5 by using 1 mol/L NaOH or HCl. After 30 min, their flocculating activities were measured at room temperature by the flocculating test described by Guo *et al*.^25^. It turned out that flocculating activity of the EPS was stable in a pH range of 6.5–9.5, which achieved greater than 85% and peaked at pH 7.5. Beyond this pH range, flocculating activity decreased gradually (Figure. S6). Similar study of a biopolymers produced by *Bacillus subtilis* F9 showed that it was stable in a pH range of 3–8^19^. With regard to thermal stability, the EPS samples were placed at 4, 10, 20, 30, 40, 50, 60, 70, 80, 90, 100, 110, and 120 °C for 30 min, respectively, and then their flocculating activities were measured. Results showed that when pH values of the EPS samples were in the range of 6.5–9.5, the EPS was thermo-stable and retained more than 85% flocculating activity in a temperature range of 10–80 °C, and 80% or more could still be achieved after heating the EPS for 30 min at 90–120 °C. Literatures reported that in temperature range of 30–120 °C, the polysaccharide chains can remain extended and exposed its flocculating sites, and ensure the flocculating activity^24^, such as the biopolymers from *Bacillus subtilis* F9, remained 89% of its flocculating activity at 100 °C^19^. Above thermo-stable characteristics further suggested that the main backbone of the EPS in this study was polysaccharides, similar information was achieved in case of biopolymers from hydrolysates of corn stover^18^.

Infrared spectrum of the EPS in Figure. S7 displayed a stretching peak at 3430 cm^−1^ on the characteristic of –OH and –NH_2_^15^, which was in accordance with that of NOC-1 (mainly glycoprotein) produced by R. *erythropolis* in a standard culture medium^26^. The peak around 1632 cm^−1^ may be assigned to the –CO stretching in –CONH_2_ group, and the peaks around 1080 cm^−1^ were characteristic of C–O groups, indicating the presence of carboxyl groups in the EPS molecular chains. The peak detected at 1405 cm^−1^ could be attributed to the symmetric stretching of the –COO^−^ group^20^, which could provides more adsorption sites for particle attachment, and thus, particles can be adsorbed to the long molecular chain of the EPS^27^. The behavior of the wide peaks in 1080–1405 cm^−1^ range and at 545 cm^−1^, represented the stretch vibration of C–O, which indicated the presence of polysaccharides. In summary, FTIR spectrum showed the presence of hydroxyl, amide, and carboxyl groups in the EPS structure, which were the typical functional groups of polysaccharides, and were preferred for flocculation.

### Comparison of ADSW treatment by the EPS with other flocculants

**Figure 1:**
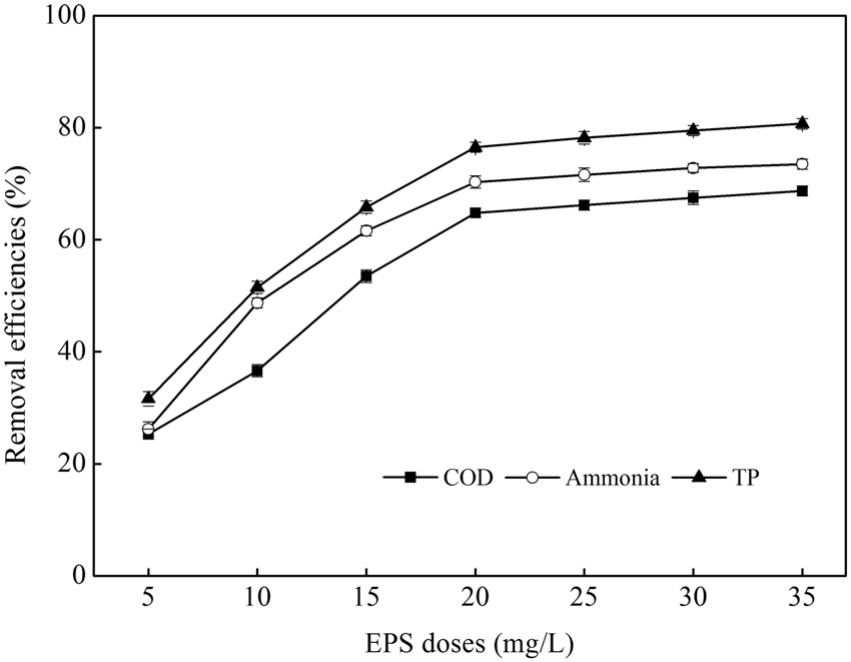
Effects of EPS doses on ADSW treatment.

**Figure 2:**
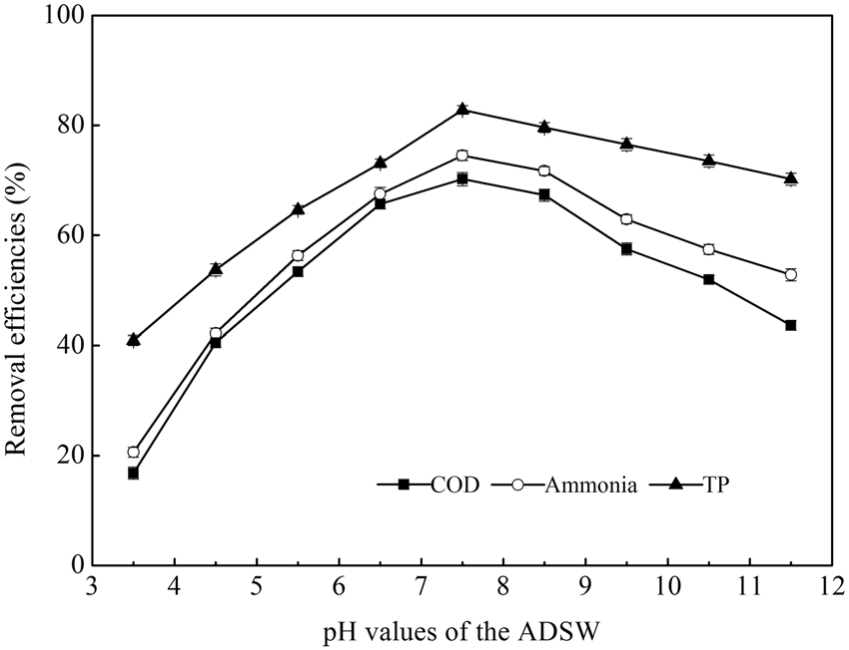
Effects of solution pH on ADSW treatment.

**Figure 3:**
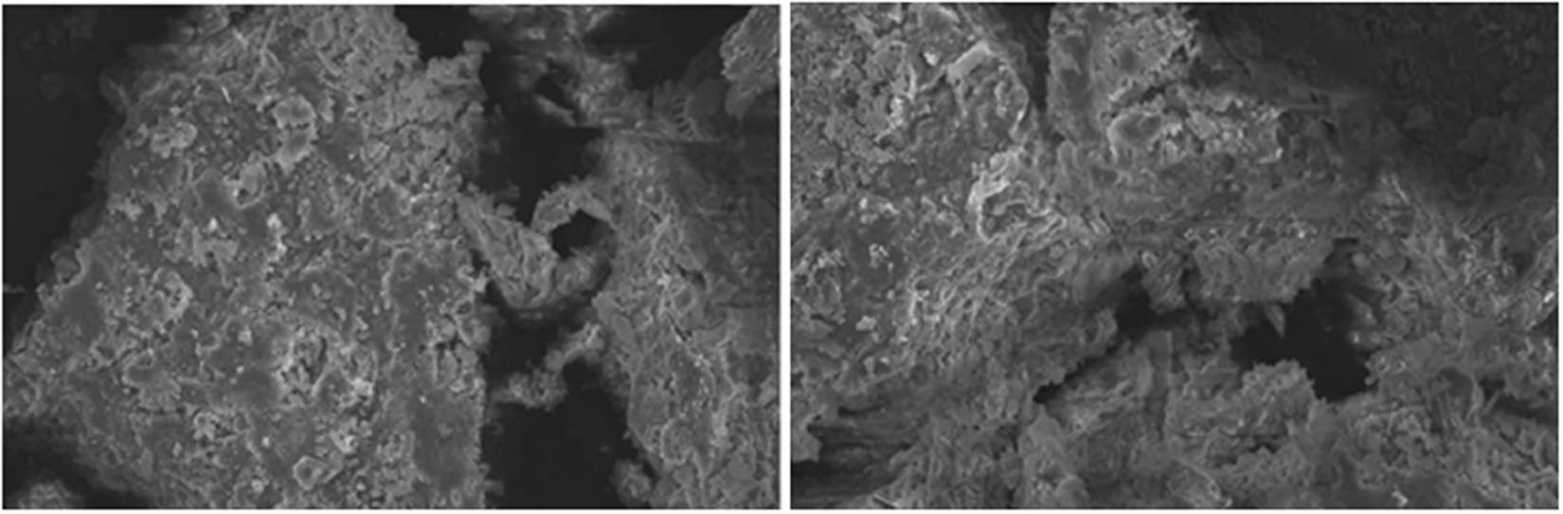
Morphology change of flocs before (left) and after (right) flocculation by EPS.

Figure 1 described that the EPS can remove COD, ammonia, and TP effectively with its doses varying from 5 to 35 mg/L. When the increasing EPS achieved to 20 mg/L, removal efficiencies increased rapidly to maximum values of 64.8%, 70.3%, and 76.5%, respectively, while the increasing EPS above 20 mg/L had negligible effects on the increasing in removal efficiencies. This may be attributed to the formation of aggregates at higher solid/liquid ratios or to precipitation of particles^25^. Figure 2 showed that this EPS performed well in solution pH range of 6.5–8.5, and the maximum removal efficiencies of 70.2%, 76.5% and 82.8% for COD, ammonia, and TP were observed when the solution pH value was adjusted to 7.5. Figure 3 showed that there was a large amount of relatively sparse gaps between flocs in the wastewater before flocculating by the EPS, after the flocculation process, large flocs were formed and were showed as a tightly structure, which were easy to be settled down, and further enhancing the removal of COD, ammonia, and TP from ADSW.

Chemicals including FeCl_3_, Al_2_(SO_4_)_3_, and PAC were often applied in pretreatment of ADSW to improve the performance of ecological processes in China, however, only one or two publications associating EPS and ADSW. Most of them dealt with mineral suspensions (e.g. kaolin), surface water (e.g. drinking water), sometimes sediments and industrial sludge. By comparing with chemical flocculants in the pretreatment of ADSW (Figures. S8–10), we can assess the processing performance by the EPS. Table 1 presented a comparison of removal efficiencies using the EPS with FeCl_3_, Al_2_(SO_4_)_3_, and PAC at their optimal doses and pH values. When ADSW pH was adjusted, flocculants, including the EPS, FeCl_3_, Al_2_(SO_4_)_3_, and PAC, were separately added into ADSW at their optimal doses of 20 mg/L, 30 g/L, 30 g/L, and 15 g/L, respectively. After conditioning, COD, ammonia, and TP removal efficiencies by the EPS were 70.2%, 76.5% and 82.8%, respectively, which were much better than FeCl_3_, Al_2_(SO_4_)_3_, but slightly poorer than PAC.

**Table 1:** Results of ADSW treatment by different flocculants.

Flocculants	Optimal dose(g/L)	Optimal pH	COD(mg/L) (RE)	Ammonia(mg/L) (RE)	TP(mg/L) (RE)
Blank	—	—	1065	828	24
FeCl_3_	30	6.5	462.2 (56.6%)	390.0 (52.9%)	11.2 (53.5%)
Al_2_(SO_4_)_3_	30	6.5	484.6 (54.5%)	399.9 (51.7%)	11.4 (52.4%)
PAC	15	7.5	270.5 (74.6%)	179.7 (78.3%)	3.8 (84.1%)
EPS in this study	0.02	7.5	317.4 (70.2%)	194.6 (76.5%)	4.1 (82.8%)

### ADSW treatment by the composite of the EPS and PAC

To understand the influence of EPS dose (*x*_1_), PAC dose (*x*_2_), pH value (*x*_3_), and agitation speed (*x*_4_) on the corresponding responses (removal efficiencies of COD (*y*_1_), ammonia (*y*_2_), and TP (*y*_3_)), the experiments were designed using RSM based on the performance of the EPS and PAC in ADSW treatment. Following equations represent empirical relationship in the form of quadratic polynomial between the removal efficiencies and factors  *x*_1_ − *x*_4_.

1 $$\begin{array}{ccc}{y}_{1} & = & 92.01+1.12{x}_{1}+4.45{x}_{2}-1.28{x}_{3}-2.41{x}_{4}-3.66{x}_{1}{x}_{2}\\  &  & -4.15{x}_{1}{x}_{3}-1.23{x}_{1}{x}_{4}-4.22{x}_{2}{x}_{3}+4.79{x}_{2}{x}_{4}-2.07{x}_{3}{x}_{4}\\  &  & -6.24{x}_{1}^{2}-2.13{x}_{2}^{2}-1.56{x}_{3}^{2}-1.62{x}_{4}^{2}\end{array}$$

2 $$\begin{array}{ccc}{y}_{2} & = & 89.64-0.44{x}_{1}+4.35{x}_{2}+0.26{x}_{3}-0.31{x}_{4}-3.26{x}_{1}{x}_{2}\\  &  & +4.33{x}_{1}{x}_{3}+0.25{x}_{1}{x}_{4}-3.74{x}_{2}{x}_{3}+0.25{x}_{2}{x}_{4}-0.47{x}_{3}{x}_{4}\\  &  & -1.67{x}_{1}^{2}-6.34{x}_{2}^{2}-4.27{x}_{3}^{2}-2.28{x}_{4}^{2}\end{array}$$

3 $$\begin{array}{ccc}{y}_{3} & = & 93.37+0.46{x}_{1}+5.21{x}_{2}+1.39{x}_{3}+0.45{x}_{4}-3.62{x}_{1}{x}_{2}\\  &  & +6.21{x}_{1}{x}_{3}+1.36{x}_{1}{x}_{4}-4.33{x}_{2}{x}_{3}+2.26{x}_{2}{x}_{4}+2.38{x}_{3}{x}_{4}\\  &  & -1.69{x}_{1}^{2}-5.42{x}_{2}^{2}-2.43{x}_{3}^{2}-3.17{x}_{4}^{2}\end{array}$$

Statistical testing of these models was performed with the Fisher’s statistical method for analysis of variance (ANOVA)^25^. Results of ANOVA analysis in terms of coded variables for COD, ammonia, and TP indicated that all the final models were significant at 95% confidence level with values of ‘*Prob* > *F*’ (<0.0001) less than 0.05. Meanwhile, as presented in Table 2, *p*-value (0.0017, 0.0121, and 0.0087 for the three empirical models) for the lack of fit test was less than 0.05, suggesting that there were almost no systematic variation unaccounted in the models. As an evaluation of the model’s overall performance, all the determination coefficient *R* ^2^ values in this study were relatively high (0.9422, 0.9745, and 0.9668), indicating a good agreement between the model predicted and the experimental values. Meanwhile, adjusted *R* ^2^ values were 0.9405, 0.9667, and 0.9673 for the three models, which were not significantly different with the determination coefficient *R* ^2^ values, showed that there were almost no chances that insignificant terms have been included in the model. Moreover, values of 14.82, 17.66, and 16.39 of adequate precision (AP) were found to be desirable for all models (AP greater than 4 is desirable).

**Table 2:** ANOVA results for the three responses.

Responses (RE, %)	Item	Sum of squares	Degrees of freedom	Mean square	*F*-value	Prob > *F*	p-value	*R* ^2^	Adjusted *R* ^2^	AP
COD	Model	2866.47	14	205.53	19.28	<0.0001^a^				
	Lack of fit	121.63	10	14.89	49.72		0.0017^a^	0.9422	0.9405	14.82
ammonia	Model	2912.31	14	213.38	26.53	<0.0001^a^				
	Lack of fit	108.83	10	10.44	6.26		0.0121^a^	0.9745	0.9677	17.66
TP	Model	2846.25	14	205.12	20.74	<0.0001^a^				
	Lack of fit	142.18	10	13.37	13.28		0.0087^a^	0.9668	0.9673	16.39

^a^Significant

AP: adequate precision; *R* ^2^: determination coefficient; Adjusted *R* ^2^: adjusted determination coefficient.

Significance testing for the coefficient of Eqs (1)–(3) whose variables in terms of coded factors was listed in Table 3. For all the parameters, in the linear terms, pH value of the ADSW was significant, due to its affection to the density of hydrogen ions (H^+^), the dominant ionic species of ammonia, and the surface charge of the EPS, in the removal of COD, ammonia, and TP, which has been proved in our previous study^13^. Among the higher order effects, the quadratic terms of EPS and PAC dose were significant and unique. For EPS attributing to the absorption bridging action, its flocculating mechanism, could promote the flocculation by influence the size and density of flocs. Particles adsorbed to an EPS molecular chain, and they could be adsorbed simultaneously to other chains, leading to the formation of three-dimensional flocs, which were capable of rapid settling. A deficient dose of the EPS cannot make all the particles flocculated to become compact flocs. Conversely, an excessive EPS concentration may prevent the small flocs from growing into big ones, due to the increasing electrostatic repulsion between the excessive EPS chains, which enhancing the difficulty of pollutants removal^28^. PAC was significant for the reason that excessive dose of PAC led to the stabilization of colloid suspension which could disaggregate the flocs again.

**Table 3:** Significance of quadratic model coefficient of for the three responses.

Responses (RE, %)	Independent variables	Coefficient estimate	Degrees of freedom	Standard error	Prob > F
COD	*x*_3_	6.92	1	0.94	<0.0001 ^a^
	*x*_1_ × _2_	−4.75	1	1.63	0.0112 ^a^
	*x*_1_ × _3_	−4.25	1	1.63	0.0108 ^a^
	${x}_{1}^{2}$	−6.47	1	1.28	<0.0001 ^a^
	*x*_2_^2^	−16.22	1	1.28	<0.0001 ^a^
ammonia	*x*_3_	6.00	1	0.81	<0.0001 ^a^
	*x*_1_ × _2_	−5.00	1	1.40	0.0045 ^a^
	${x}_{1}^{2}$	−8.07	1	1.10	<0.0001 ^a^
	${x}_{2}^{2}$	−16.57	1	1.10	<0.0001 ^a^
TP	*x*_3_	5.08	1	0.90	<0.0001 ^a^
	*x*_1_ × _2_	−3.50	1	1.57	0.0023 ^a^
	*x*_1_ × _4_	5.25	1	1.57	0.0204 ^a^
	${x}_{1}^{2}$	−12.50	1	1.23	<0.0001 ^a^
	${x}_{2}^{2}$	−14.50	1	1.23	<0.0001 ^a^

^a^Significant.

**Figure 4:**
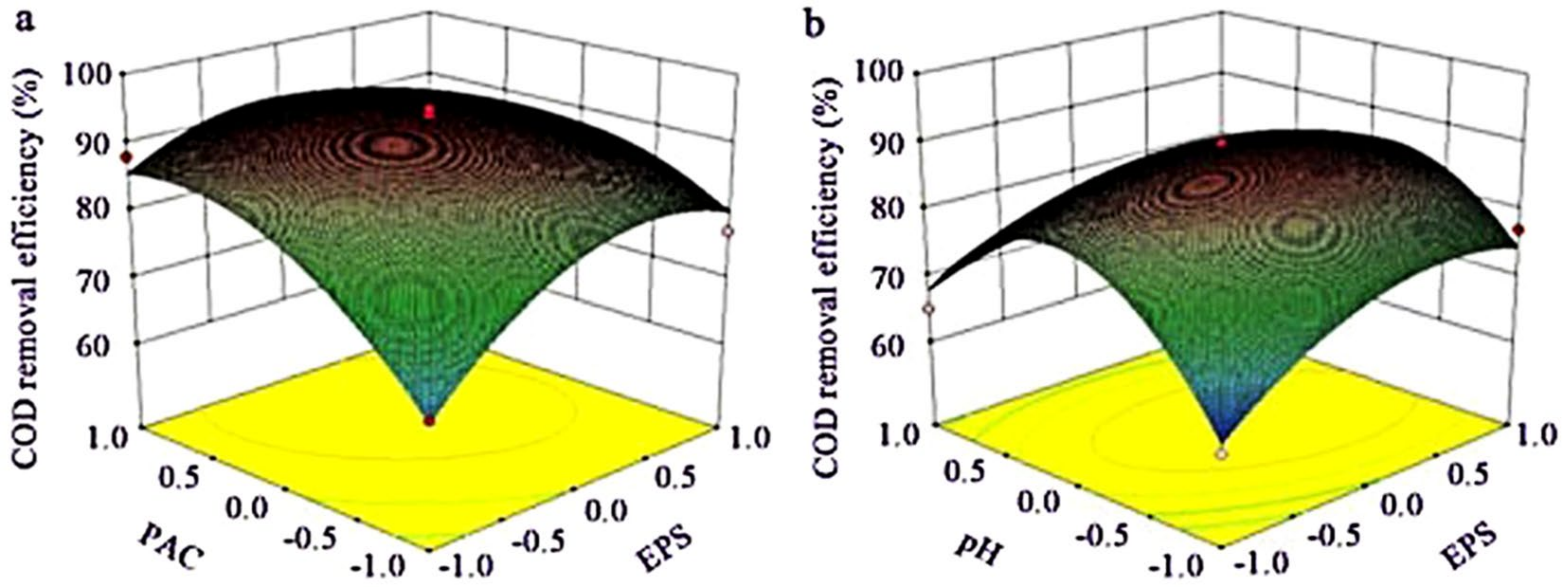
Surface graphs of COD removal efficiency showing the effect of variables: (**a**) PAC-EPS; (**b**) EPS-pH.

**Figure 5:**
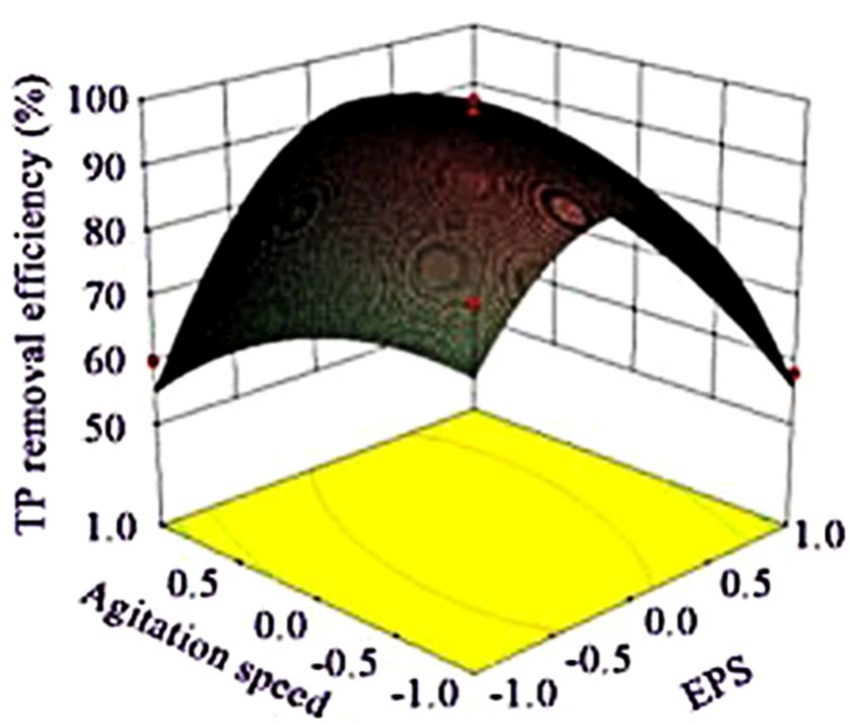
Surface graphs of ammonia removal efficiency showing the effect of variables: PAC –EPS.

**Figure 6:**
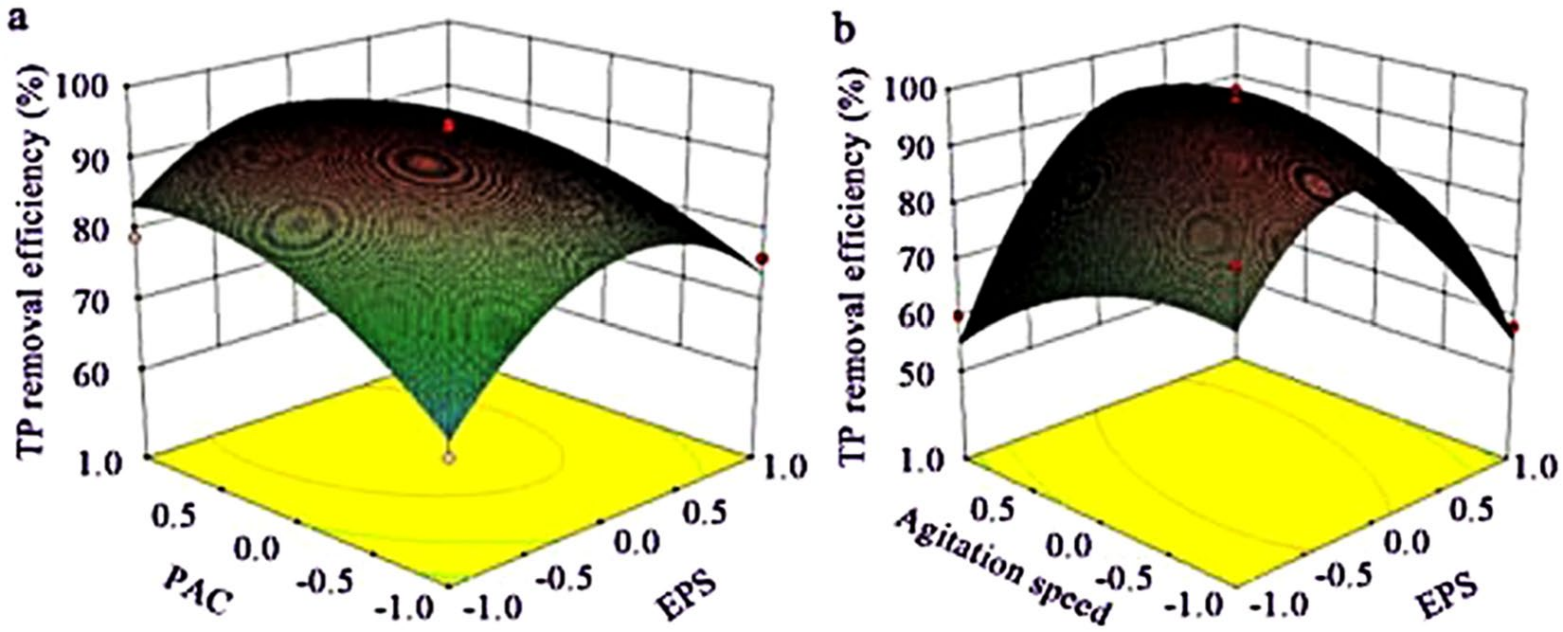
Surface graphs of turbidity removal efficiency showing the effect of variables: (**a**) PAC -EPS; (**b**) EPS-agitation speed.

Figures. 4–6 depicted the interaction terms with significant effect. From Figure. 4a, the EPS has obvious effect on COD removal in composite process, COD removal enhanced as PAC added and gets to the peak ultimately, and this target obviously became easier when EPS was kept at central level, suggested that PAC was a proper flocculant which could produce a positive effect on COD removal at low level of EPS for compounding^29^. From Figure. 4b, COD removal efficiency can get to the anticipant value at a small quantity of EPS when pH was higher than 7.5 approximately. Figure. 5 showed that ammonia removal was enhanced when the composite of PAC and EPS was used, and it is predicted that, at a low level of PAC, ammonia removal efficiency was enhanced as the EPS added and it can get to the peak ultimately. Figure. 6a showed that EPS has obvious effect on the improvement of TP removal in composite process, and this was similar with that conducted by Liu *et al*., in which the composite of a biopolymer and PAC showed the same tendency in landfill leachate treatment. Figure. 6b provided evidences that suitable agitation speed can improve particles settling under the appropriate EPS dose and pH value. Top speed of stir could be used as a rough measure of mixing effectiveness, based on the reasoning that a higher speed creates greater turbulence, and greater turbulence leads to better mixing^30^, however, the time that forming the flocs needed were different in each kinds of flocculating conditions, and a greater turbulence environment had a more negative impact on flocs forming. Therefore, the suitable agitation speed was important for pollutants removal from wastewater.

According to the target values of the three responses, removal efficiencies of COD, ammonia, and TP were all 100%, the optimal condition calculated from the regression equations were EPS of 16 mg/L, PAC of 12 g/L, pH of 7.5, and agitation speed of 200 r/min. Under this optimal condition, COD, ammonia, and TP removal rates appeared as 91.6%, 90.8%, and 92.5%, respectively.

## Discussion

The EPS showed good performances in ADSW treatment, after conditioned by this EPS with a dose of 20 mg/L and a pH condition of 7.5, removal efficiencies of COD, ammonia, and TP reached 70.2%, 76.5% and 82.8%, respectively, which were much better than the ones obtained with chemicals. Due to the high molar weight and polar groups, the EPS can supply ample binding sites, strong bridging, and strong van der Waals forces for suspended solids in ADSW, and thus, suspended solids can be adsorbed to EPS molecular chains to settle rapidly, thus far improved the removal of COD, ammonia, and TP from ADSW^31^. Furthermore, the organic materials can be reacted with the polar groups of the EPS molecular chains, which could promote the removal of organic pollutants. The removal of COD, ammonia, and TP by the EPS was highly dependent on pH value of ADSW. In strong acid and alkali environments, the removal was poor, because the electrostatic repulsion of negatively charged particles was increased due to the raised negatively charge density with the increasing pH of above 8.5, which leading to the depression of the removal efficiencies. On the contrary, the increased H^+^ concentration with the decreasing in water’s pH value of below 6.5 promoted the sharing between the excess H^+^ and some functional groups of the EPS molecular chains, which reduced the removal efficiencies as well.

Compound use of this EPS and PAC was testified to be feasible in ADSW treatment. Enhanced flocculation mechanisms of the component were elaborated, according to the classic theory of double electrical layer, and PAC compressed double electrical layers of particles by charge neutralization. Therefore, relatively more compact primary flocs were accumulated to small granules as a result of electrostatic attraction between cationic electrolyte and negatively charged surfaces^32^. Moreover, for the EPS attributing to the absorption bridging action, more stable particles adsorbed onto one EPS molecular chain, and they could be adsorbed simultaneously by other chains, leading to formation of three-dimensional flocs, which further promoted pollutants removal^28^.

## Materials and Methods

### Microorganism strain isolation and identification

EPS-producing strain was enriched from activated sludge by using LB medium. The enrich process was given as follows: for isolation of high-ammonia resistant strain, an activated sludge sample was collected from Hangkonggang wastewater treatment plant, Sichuan province, China, and total of 1 mL of this sludge sample was serially diluted with distilled water (10^1^–10^10^ folds). Subsequently, 1 mL of each dilution was inoculated in LB mediums supplemented with 100 mg/L of ammonia, and was incubated at 35 °C in an incubator till substantial microbial growth. Large and viscous colonies were then inoculated in LB mediums supplemented with 200 mg/L of ammonia, and were incubated at the same temperature to enrich strains, after 10 cycles of enrichment in the same procedure, a total of 26 morphologically different isolates were obtained, which can grow well on the LB mediums with addition of 1000 mg/L of ammonia. In these 26 colonies, 8 large and viscous ones were chosen and individually inoculated on a reciprocal shaker at 150 r/min and 35 °C for 24 h in a culture medium consisted of distilled water 150 mL, urea 0.75 g, glucose 3 g, K_2_HPO_4_ 0.6 g, and KH_2_PO_4_ 0.3 g. After cultivation for 60 h, flocculating activities of the broth obtained by the 8 strains were individually measured by the flocculating test described by Guo *et al*.^25^ and the ones with the highest flocculating activity of 90.6%, named G106, was selected to produce EPS.

Cell forms and colony characteristics of the strain G106 on nutrient agar was observed with bio-microscope (CX31, Olympus, Japan), and it’s physiological and biochemical characteristics were identified according to Bergey’s Manual of systematic bacteriology. The 16 S rRNA gene fragment was then amplified using individual bacterial colony PCR. PCR amplification was carried out using forward primer (5′-GAG AGT TTG ATC CTG GCT CAG-3′) and reverse primer (5′-CTA CGG CTA CCT TGT TAC GA-3′), and was run on a MyCycler thermal cycle (Bio-Rad, USA) using cycling conditions as follows: 94 °C for 4 min; followed by 30 cycles of 94 °C for 90 s, 55 °C for 60 s, 72 °C for 90 s; followed by 72 °C for 7 min, and ended at 4 °C.

### EPS production and its physic-chemical characteristics

Anaerobically digested swine wastewater (ADSW) from a local piggery farm in Sichuan province, China, was selected as cheap fermentation medium to produce EPS. The concentrations of chemical oxygen demand (COD), ammonia, and total phosphorus (TP) in the ADSW were 1065, 828, and 24 mg/L, respectively. pH value of the ADSW was 6.7. For EPS production, the strain G106 was inoculated directly in ADSW medium, and was incubated on a reciprocal shaker at 150 r/min and 30 °C. In EPS production process, effects of initial pH value of the ADSW medium, phosphate salts additions, carbon additions, and nitrogen additions were determined. After fermentation for 60 h, the broth was collected, from which EPS can be extracted by using the methods proposed by Aljuboori *et al*.^17^, with slight modification. Briefly, the fermentation broth with flocculating components was centrifuged at 3000 r/min for 30 min and the supernatant was collected by discharging the cell pellets. Subsequently, the supernatant was precipitated with three volumes of cold anhydrous ethanol by incubating the mixture at 4 °C for 24 h, and the resulting precipitate collected at the same procedure (3000 r/min, 30 min) was denoted as the crude EPS. For EPS purification, the crude EPS was dissolved in deionized water followed by the addition of 10% cetylpyridinium chloride (Sigma-Aldrich, USA) with continuous stirring. After 4 h, the resulting precipitate was collected also at the same procedure (3000 r/min, 30 min) and was re-dissolved in 0.5 mol/L of NaCl solution. At last, two volumes of cold anhydrous ethanol were again added to obtain the precipitate, which was then washed three times with 75% ethanol and lyophilized to obtain the purified EPS.

Contents of total sugar, uronic acid and amino sugar of the purified EPS were determined by phenol-sulfuric acid method, carbazol-sulfuric acid method, and Elson-Morgan method, respectively^33^. Protein content was measured by the Bradford method using bovine serum albumin as the standard solution^34^. Elemental analysis of the EPS was achieved by using an elemental analyzer (PE 2400 II, Perkin Elmer Company, USA). Molar mass was determined by gel permeation chromatography (GPC) using a Hitachi L-6200 system controller (IET Ltd., USA). Functional groups of the EPS were determined using a Fourier transform infrared (FTIR) spectrophotometer (EQUINOX 55, Bruker Company, Germany). Surface morphology of the EPS was observed and elucidated by using scanning electron microscopy JEOL (JSM-6390LV, Tokyo, Japan).

### ADSW treatment by the EPS

A standard jar-test apparatus (ET-720, Lovibond, Germany) comprising six paddle rotors in 400 mL beakers was used for ADSW treatment experiments, ADSW from a local piggery farm (section “EPS production and its physic-chemical characteristics”) was chosen as the representative suspended sample. ADSW samples with volume of 200 mL were added into the beakers and their pH values were adjusted to the desired levels using 1 mol/L NaOH or HCl if necessary. Flocculants, including the EPS, FeCl_3_, Al_2_(SO_4_)_3_, and PAC, were then added into ADSW separately and the mixtures were immediately at 200 r/min for 30 min. After agitation, all the samples were allowed to stand for 30 min. Thereafter, a 50 mL supernatant from each beaker was withdrawn and filtered by 0.45 μm filter membrane to determine the residual COD, ammonia, and TP, and their removal efficiencies (RE) can be calculated according to the following equations:

4 $$\mathrm{RE}( \% )=\frac{{C}_{0}-{C}_{{\rm{e}}}}{{C}_{0}}\times 100 \% $$

where *C*_0_ and *C*_e_ were initial and final concentrations of COD, ammonia, and TP of ADSW, respectively.

### RSM experimental design, analysis and optimization

Central composite design (CCD), the standard RSM, was selected to investigate the interactions of parameters including EPS dose (*x*_1_), PAC dose (*x*_2_), pH value (*x*_3_), and agitation speed (*x*_4_). The response variables (*y*) that represented COD, ammonia, and TP removal efficiencies were fitted by a second-order model in the form of quadratic polynomial equation:

5 $$y={\beta }_{0}+\sum _{i=1}^{m}{\beta }_{i}{x}_{i}+\sum _{i < j}^{m}{\beta }_{ij}{x}_{i}{x}_{j}+\sum _{i=1}^{m}{\beta }_{ii}{x}_{i}^{2}$$

where *y* was the response variable to be modeled, *x*_*i*_ and *x*_*j*_ were independent variables which was used to determine *y*. *i* was linear coefficient and the quadratic coefficient. *β*_0_, *β*_*i*_ and *β*_*ii*_ were the offset term. *β*_*ij*_ was the term that reflect the interaction between *x*_*i*_ and *x*_*j*_. The actual design was run by the statistic software Design-expert 8.0.5 and was presented in Table 4.

**Table 4:** Coded levels for four variables framed by the Central Composite Design.

Factors	Codes	Codes levels		
		−1	0	1
EPS (mg/L)	*x*_1_	10	20	30
PAC (g/L)	*x*_2_	10	15	20
pH	*x*_3_	5.5	7.5	9.5
Agitation speed (r/min)	*x*_4_	100	200	300

All the measurements through this study were carried out in triplicates and the average values were presented (with standard error less than 5% of the mean).

## Electronic supplementary material

Supplementary information
