# Supplementary material for: Enhanced anaerobically digested swine wastewater treatment by the composite of polyaluminum chloride (PAC) and Bacillus megatherium G106 derived EPS
Source: Sci Rep. 2017 Aug 17;7:8605. doi: 10.1038/s41598-017-09044-0 (PMC5561036; doi:10.1038/s41598-017-09044-0)
Supplement: Supplementary file 1 — Supplementary information [file 41598_2017_9044_MOESM1_ESM.doc]

Electronic Supplementary Information

For

**Enhanced anaerobically digested swine wastewater treatment by the composite of polyaluminum chloride (PAC) and *Bacillus megatherium* G106 derived EPS**

**Junyuan Guo*, Yang Huang*, Cheng Chen, Yu Xiao, Jing Chen, Biyu Jian**

College of Resources and Environment, Chengdu University of Information Technology, Chengdu, Sichuan 610225, China.

* Corresponding author

Junyuan Guo, Yang Huang

E-mail: gjy@cuit.edu.cn, [huangyang@cuit.edu.cn](mailto:huangyang@cuit.edu.cn)

Tel and Fax: 86-28-8596-6913

**Figures and Tables**

**Fig.S1** Effects of phosphate salts on EPS yield and its flocculating activity

**Fig.S2** Effects of extra carbon sources on EPS yield and its flocculating activity

**Fig.S3** Effects of extra nitrogen sources on EPS yield and its flocculating activity

**Fig.S4** Effects of pH value of the wastewater medium on EPS yield and its flocculating activity

**Fig.S5** Growth curve of the EPS-producing strain

**Fig.S6** pH stability and thermal stability of the EPS

**Fig.S7** FTIR spectrum of the EPS

**Fig.S8** Effects of FeCl_3_ doses (a) and pH value (b) on ADSW treatment

**Fig.S9** Effects of Al_2_(SO_4_)_3_ doses (a) and pH value (b) on ADSW treatment

**Fig.S10** Effects of PAC doses (a) and pH value (b) on ADSW treatment

**Fig.S1** Effects of phosphate salts on EPS yield and its flocculating activity

**Fig.S2** Effects of extra carbon sources on EPS yield and its flocculating activity

**Fig.S3** Effects of extra nitrogen sources on EPS yield and its flocculating activity

**Fig.S4** Effects of pH value of the wastewater medium on EPS yield and its flocculating activity

**Fig.S5** Growth curve of the EPS-producing strain

**Fig.S6** pH stability and thermal stability of the EPS


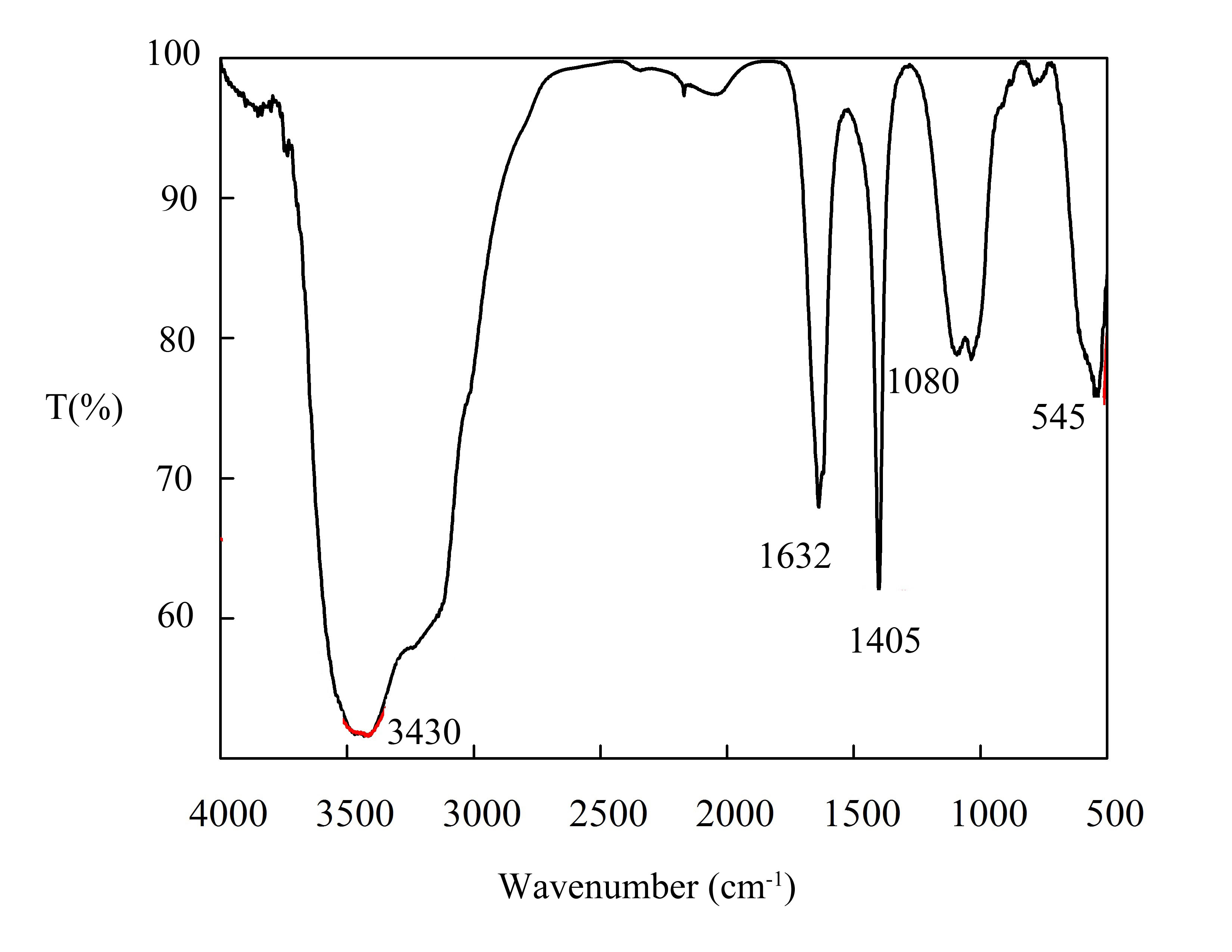


**Fig.S7** FTIR spectrum of the EPS

**Fig.S8** Effects of FeCl_3_ doses (a) and pH value (b) on ADSW treatment

**Fig.S9** Effects of Al_2_(SO_4_)_3_ doses (a) and pH value (b) on ADSW treatment

**Fig.S10** Effects of PAC doses (a) and pH value (b) on ADSW treatment
